# Vascular Protection by Ethanol Extract of* Morus alba* Root Bark: Endothelium-Dependent Relaxation of Rat Aorta and Decrease of Smooth Muscle Cell Migration and Proliferation

**DOI:** 10.1155/2018/7905763

**Published:** 2018-11-01

**Authors:** Nisha Panth, Keshav Raj Paudel, Dal-Seong Gong, Min-Ho Oak

**Affiliations:** ^1^College of Pharmacy and Natural Medicine Research Institute, Mokpo National University, Muan-gun, Jeonnam 58554, Republic of Korea; ^2^Department of Oriental Medicine Resources, Mokpo National University, Muan-gun, Jeonnam 58554, Republic of Korea

## Abstract

*Morus alba* (white mulberry) is native to the northern part of Korea and popularly used as a traditional medicine due to its numerous health benefits against human's disease. However, the possibility that* M. alba* may also affect the cardiovascular system remains unexplored. This study sought to investigate the vascular protective effects of the root bark extract of* M. alba* (MAE). Vascular reactivity was performed in organ baths using isolated rat thoracic aorta, while platelet derived growth factor (PDGF) induced proliferation and migration of vascular smooth muscle cells (VSMCs) were studied by 3-(4,5-dimethylthiazol-2-yl)-5-(3-carboxymethoxyphenyl)-2-(4-sulfophenyl)-2H-tetrazolium (MTS) and wound healing assay, respectively. MAE evoked a concentration dependent vasorelaxation following endothelium-dependent pathway. However, vessel relaxations in response to MAE were markedly reduced after endothelium removal; treatment of endothelial nitric oxide synthase inhibitor, guanylyl cyclase inhibitor, and nonspecific potassium channel inhibitor, however, was not altered by cyclooxygenase inhibitor. Furthermore, MAE also significantly blunted contractile response to vasoconstrictor agent, phenylephrine. Taken together, the current evidence revealed that MAE is a potent endothelium-dependent vasodilator and this effect was involved in, at least in part, nitric oxide cyclic-guanosine monophosphate (NO-cGMP) pathway in combination with potassium (K^+^) channel activation. Moreover, MAE inhibited proliferation and migration of VSMCs induced by PDGF. Therefore, MAE could be a promising candidate of natural medicine for preventing and controlling cardiovascular diseases linked with endothelial dysfunction.

## 1. Introduction

In global scenario, cardiovascular diseases (CVDs) are still the major cause of morbidity and mortality in developed nations, while the prevalence is rising rapidly in underdeveloped country too [1, 2]. The majority of CVDs result from complications of atherosclerosis or vascular inflammation initiated by endothelial dysfunction and leading to various pathological conditions like peripheral arterial disease, coronary heart disease, and hypertension [3]. A single intimal layer of blood vessel composed of endothelial cell release, a potent vasodilator, and nitric oxide (NO) to maintain vascular homeostasis [4, 5]. This homeostasis is maintained under normal conditions by the cardioprotective role of endothelial factors. However, toxic insults to the endothelial cell by various components like oxidative stress, abnormal cholesterol level, lipid peroxidation, and mitogen lead to endothelial dysfunction that results in defect in NO production leading to impaired endothelium-dependent vasodilation [6–8]. Below the intimal endothelial cell layer, there are vascular smooth muscle cells (VSMCs) in adventitial layer. Migration of VSMCs from tunica adventitia to tunica intima followed by proliferation at that site contributes to the growth of atherosclerotic plague and restenosis [9]. Both migration and proliferation of VSMCs are initiated by number of inducing factors such as platelet derived growth factors (PDGF) and tumor necrosis factor alpha (TNF-*α*) facilitating atheroma formation in vessel wall [10]. These days, long-term therapeutic approach to control CVDs by treatment of modern/western medicine possesses many undesirable side effects so traditional natural medicines are considered as safe and effective alternative to those synthetic drugs [11–14]. In present context, increasing health issue urged the researchers to revitalize the natural products and to alleviate health problems without harming vital functions of the body [15].

The mulberry tree (*Morus alba*) has been used for CVD prevention and treatment for more than a millennium in eastern countries, particularly in China, Japan, and Korea. The root bark of* M. alba* has been used in traditional medicine as anti-inflammatory, hypoglycemic, and antibacterial agent [16]. In addition, the root extract of* M. alba* has been reported to have anticonvulsant and antihypertensive effects in different experimental animal models (mice, rats, guinea pigs, and dogs) [17]. Pharmacological analysis showed that flavonoids, coumarins, and phenols, such as rutin and quercetin, are the principal bioactive constituents in* M. alba* root bark [18, 19]. Rutin and quercetin have been demonstrated to induce significant vasorelaxation [20, 21]; therefore, we hypothesized that* M. alba* root extract may exert vasorelaxation activity. Previous research has demonstrated that* M. alba *leaf extract exerts vasorelaxant effect [22]; however, to the best of our knowledge, the effect of* M. alba* root extract on the changes of vascular tone has not been studied before.

The aim of the present study was to investigate whether root bark extract of* M. alba* (MAE) causes endothelium-dependent vasorelaxation on rat aortic rings and to characterize the underlying mechanisms of action. Likewise, the effect of K^+^ channel blockers on MAE*-*induced vasorelaxation was also investigated. In addition, the effect of MAE on VSMCs proliferation and migration was also examined.

## 2. Materials and Methods

### 2.1. Plant Extract

*M. alba* was collected from the southern part of the Jeonnam province of the Republic of Korea. The dried root bark (20g) was extracted with hot 70% ethanol for four hours. After subjecting the crude extract to filtration, the resulting ethanol extract was evaporated using a rotatory evaporator to obtain a concentrated extract. Finally, the powder of MAE was collected after freeze drying to yield total extract power of 2.7 g. For vasorelaxation study, MAE was freshly dissolved in Krebs bicarbonate solution to get desired concentrations.

### 2.2. Vascular Reactivity Study

Male Sprague-Dawley rats of 8 weeks were purchased from Central Lab. Animal Inc. (Seoul, Republic of Korea). The animals were acclimatized under controlled environments (room temperature: 23±3°C, relative humidity: 55±15%, 12-hour dark and light cycle and 10-20 times ventilations per hour). Rat received commercially available standard diets (Cargill Agri Purina, Inc., Seongnam, Republic of Korea) and water. All procedures were approved by institutional guidelines for the ethical care of animals.

Rats were anesthetized with pentobarbital (120 mg kg^−1^, i.p.) and the descending thoracic aorta was excised and immersed in chilled Krebs–Henseleit solution of the following composition (mM): NaCl 119, KCl 4.7, CaCl_2_ 1.5, NaHCO_3_ 25, KH_2_PO_4_ 1.2, MgSO_4_ 1.1, and D-glucose 10) and bubbled with 95% O_2_ + 5% CO_2_ (pH 7.4). The thoracic aorta was carefully cleaned of adhering fat and connective tissue and cut into rings about 2-3 mm in length. Aortic rings were then mounted in standard organ chambers containing 10ml Krebs–Henseleit at 37°C and continuously aerated with 95% O2 +5% CO2 mixture. The tissues were maintained under tension of 2 g and equilibrated for a period of 90 minutes before initiating experimental protocols and the solution was replaced every 15 minutes. Changes in tension were recorded by isometric transducers connected to a data acquisition system.

After an equilibration period of 90 minutes, the vessels were maximally contracted with phenylephrine (1 *μ*M) to test their contractile capacity. The presence of functional endothelium was assessed in all preparations by the ability of acetylcholine (10 *μ*M) to induce more than 80% relaxation of rings precontracted with phenylephrine (1 *μ*M). In some experiments, endothelium was removed gently rubbing the intimal surface of the blood vessel with a pair of forceps; vessels were denuded of functional endothelium when there was the absence of acetylcholine relaxant activity.

### 2.3. Characterization of the Relaxant Effect of Polyphenolic Compound

The vasorelaxant activity of MAE was measured in aortic rings with or without functional endothelium precontracted with phenylephrine (1uM) (i.e., 80% of maximal response obtained in vessels with functional endothelium). When the contraction to phenylephrine had stabilized, cumulative concentrations of MAE were added. In some experiments, rings were induced with inhibitors: L-NA (N*ω*-nitro-L-arginine, 10 *μ*M: a competitive inhibitor of NO synthase), ODQ (1*H*-[1,2,4] oxadiazole [4,3-a] quinoxalin-1-one, 10 *μ*M: an inhibitor of guanylyl cyclase), TEA (tetraethylammonium, 1mM: nonspecific K^+^ channel inhibitor), and INDO (indomethacin, 10 *μ*M: an inhibitor of cyclooxygenase), added to the bath 30 min prior to the phenylephrine addition.

### 2.4. Cell Culture

VSMCs were obtained from the Bio-Whittaker (San Diego, CA). Media were supplemented with 15% fetal bovine serum (FBS), penicillin (100 U/mL), streptomycin (100 U/mL), fungizone (250 *μ*g/mL), and L-glutamine (2 mM). Early passages of VSMCs were grown to 80–90% confluence and used throughout the experiment. In a typical experiment, the cells were starved in serum-free culture medium containing 0.1% FBS for 24 hours.

### 2.5. VSMCs Proliferation Assay

VSMCs proliferation assay was assessed by using 3-(4,5-dimethylthiazol-2-yl)-5-(3-carboxymethoxyphenyl)-2-(4-sulfophenyl)-2H-tetrazolium (MTS) assay as described previously [23, 24]. VSMCs seeded into 96 well plates were treated with PDGF-BB (20 ng/ml) and MAE at various concentrations for additional 24 hours. Then, MTS solution was added for 4 hours and absorbance was measured at 540 nm.

### 2.6. VSMCs Migration Assay

Cell migration assay was performed in IncuCyte (Essen Bioscience) as described previously [23]. Briefly, 4 × 10^5^ cells were plated on the 96-well ImageLock plates (Essen BioScience) and incubated until confluent monolayer is formed. Wounds were made by using the 96-pin wound maker and the cells were treated with and without PDGF-BB (20 ng/ml) for 48 hours in the presence and absence of increasing concentrations of MAE. Cell migration was monitored in real time by IncuCyte software.

### 2.7. Statistical Analysis

Prism 5.03 (Graph Pad Software Inc., San Diego, CA, USA) was used to perform statistical data analysis. All data were presented as mean ± SEM. Groups were compared by using ANOVA followed by Turkey's multiple comparison test and Bonferroni posttests. P-values ≤ 0.05 were considered statistically significant.

## 3. Results

### 3.1. MAE Induces Endothelium-Dependent Relaxation in Rat Aorta and Prevents Contractile Response in Rat Aorta

**Figure 1:**
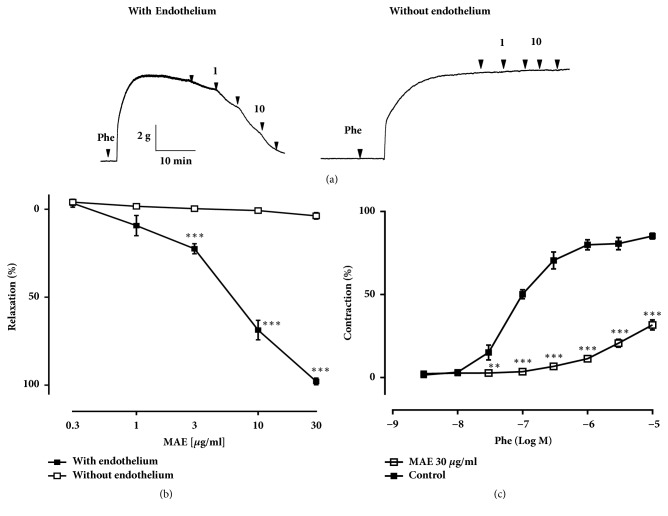
Dose response plot of vascular relaxation to phenylephrine in rings with and without endothelium incubated with MAE. Representative original tracing (a) and corresponding cumulative data (b). Concentration-contraction curves of the phenylephrine on MAE incubated mice aorta rings with endothelium (c). The contraction response is expressed as the percentage of the phenylephrine-induced contraction. The relaxation response is expressed as the percentage relaxation of the phenylephrine-induced contraction (100% represent complete relaxation). Values are the mean ± SEM (n=5). *∗*p<0.05, *∗∗*P<0.01, and *∗∗∗*P<0.001, significant difference compared with endothelium-denude rings and control.

MAE showed endothelium-dependent relaxations in rat aorta, but only nominal response was seen in those without endothelium (Figures 1(a) and 1(b)). The effective dose 50 (ED_50_) value of the vasorelaxant effect of MAE was 6.13 ± 0.67 *μ*g/mL. Moreover, the likelihood that MAE affect contractile response was studied. MAE exposed to rat aorta rings for 30 minutes prior to the addition of increasing concentrations of phenylephrine notably weaken contractions (Figure 1(c)). These data indicate that MAE may abolish contractile response by stimulating the formation of NO.

### 3.2. MAE Induces Endothelium-Dependent NO-Mediated Relaxation via the NO-sGC Pathway

**Figure 2:**
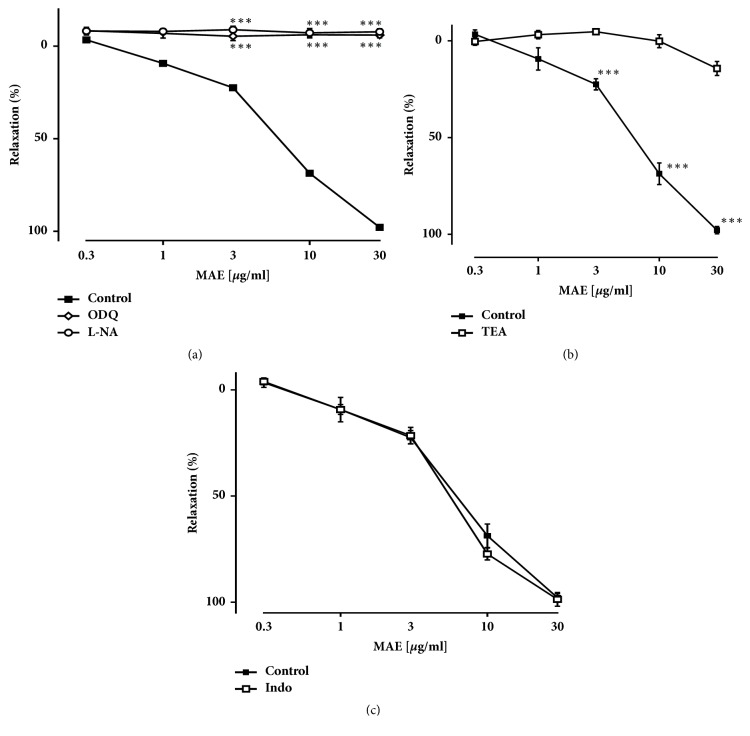
Inhibitory effect of concentration-relaxation curve on the phenylephrine-induced contraction with inhibitors L-NA 10*μ*M (a), ODQ 10*μ*M (a), TEA 1mM (b), and indomethacin 10*μ*M (c) preincubated 30 minutes prior to phenylephrine. The relaxation response is expressed as the percentage relaxation of the phenylephrine-induced contraction (100% represent complete relaxation). Values are the mean ± SEM (n=5). *∗*P<0.05, *∗∗*P<0.01, and *∗∗∗*P<0.001, significant differences compared with control.

Since previous investigations have shown activation of NO/cGMP pathway involving potassium channel activation causes vasorelaxation in rat aorta [25], the role of this pathway in the MAE-induced relaxation was determined in our experiment. Relaxation to MAE in endothelium intact rings was halted by N*ω*-nitro-L-arginine (L-NA, a competitive inhibitor of NO synthase) and 1*H*-[1,2,4] oxadiazolo [4,3-a] quinoxalin-1-one (ODQ, an inhibitor of guanylyl cyclase) (Figure 2(a)). In addition, relaxation to MAE was abolished by tetraethylammonium (TEA, nonspecific K^+^ channel inhibitor) (Figure 2(b)). However, indomethacin (INDO, an inhibitor of cyclooxygenase) did not affect relaxation caused by MAE (Figure 2(c)). These findings indicate that endothelium-dependent relaxation by MAE may follow nitric oxide cyclic-guanosine monophosphate (NO-cGMP) pathway including potassium channel activation.

### 3.3. MAE Inhibits VSMCs Proliferation and Migration

**Figure 3:**
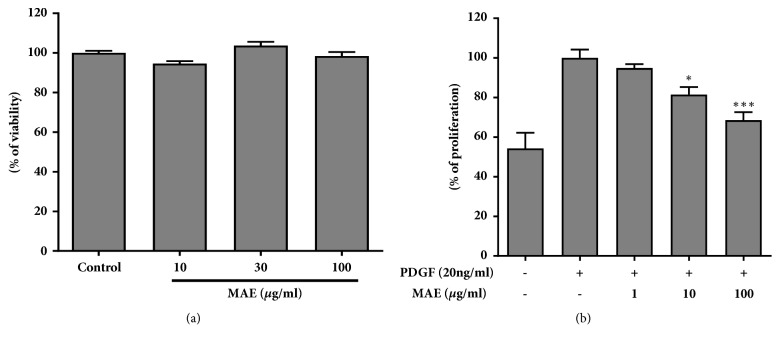
Effect of MAE on HASMCs viability and proliferation. (a) Serum starved HASMCs were pretreated with indicated concentrations of MAE for cytotoxicity. Values are expressed as mean ± SEM (n=5). (b) Cell proliferation was measured by MTS assay on PDGF-BB induce HASMCs with or without MAE. Values are expressed as mean ± SEM. *∗P*<0.05; *∗∗∗P*<0.001, significant difference versus control (PDGF-BB alone).

MAE inhibit proliferation induced by PDGF-BB dose dependently at concentrations 1, 10, and 100 *μ*g/ml (Figure 3(b)). MAE at dose of 1, 10, and 100 *μ*g/ml reduce the proliferation rate to 97%, 90%, and 79% of the control (PDGF-BB treated without MAE), respectively. We also performed the cytotoxicity assay to confirm that the inhibitory effects were not due to toxicity to the cells. MAE had no deleterious effect on the cell viability up to 100 *μ*g/ml (Figure 3(a)).

**Figure 4:**
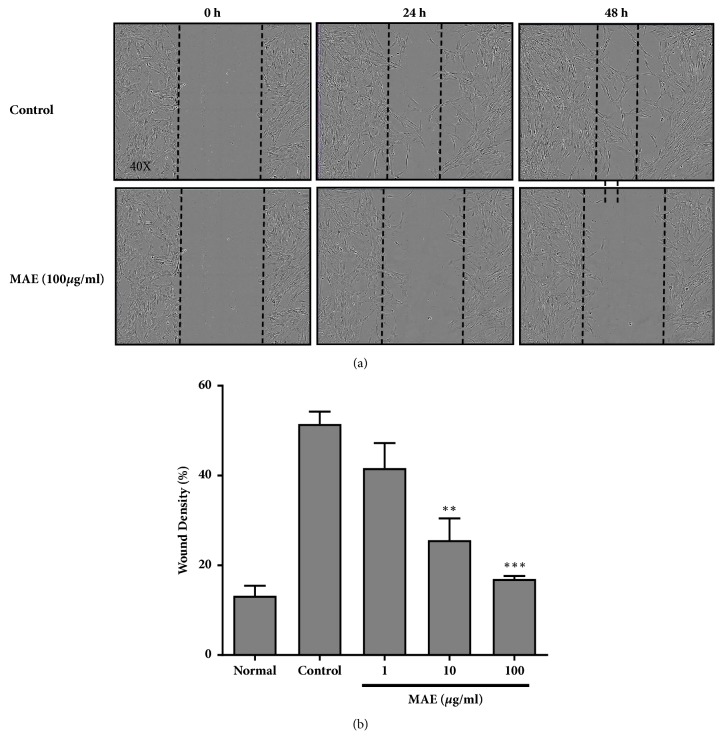
A straight wound was made on confluent VSMCs cells in 96-well plates by a commercial Wound Maker. After washing VSMCs with PBS, cells were treated with or without MAE and/or PDGF-BB and incubated in IncuCyte machine for 48 hours. Photographs of the wounded area were scanned immediately at time 0 hours and later at 24 hours and 48 hours after injury (a). The wound density in the migration zone after 48 hours was calculated in terms of percentage with respect to initial wound area filled by migrated VSMCs. Graphs are presented as mean ± SEM. *∗∗P*<0.01; *∗∗∗P*<0.001, significant difference versus control (PDGF-BB alone).

MAE at various concentration doses dependently reduced PDGF-BB induced VSMCs migration for 48 h after wound making (Figure 4(a)). MAE at concentrations 1, 10, and 100 *μ*g/ml showed the remarkable inhibition of healing (41.66%, 25.7%, and 16.96%), respectively, compared with control at 48 hours (Figure 4(b)).

## 4. Discussion

In this study, we demonstrate the first finding that the endothelium-dependent vasorelaxation to MAE may result from an increased formation of NO by endothelial cells and seems to involve the activation of tetraethylammonium-sensitive K^+^ channels.

Our result is in agreement with a previous study where the aqueous extract from leaves of* M. alba* induced vasorelaxation in rat aorta [22]. Although* M. alba* root extract has been evaluated for hepatoprotective, neuroprotective activity [23] and analgesic and anti-inflammatory activity [24], to the best of our knowledge, the vasoprotective activity of* M. alba* root bark extract (MAE) has not been studied directly before. The present study has reported for the first time that MAE indeed evoke vasorelaxation in endothelium intact rat aorta rings dose dependently. Conversely, vasorelaxation elicited by MAE was all abolished by mechanical denudation of endothelium and endothelium treated with L-NAME, an inhibitor of nitric oxide synthase. This study demonstrates that MAE may stimulate the production of NO in endothelial cells and that NO might be involved in the subsequent cyclic-GMP accumulation and endothelium-dependent relaxation [25, 26]. Accumulating evidence has indicated that NO induces vascular smooth muscle relaxation through the activation of soluble guanylyl cyclase, resulting in the accumulation of cyclic-GMP [25–27]; this finding is supported by the observation in the present study, where MAE-induced relaxation was all abolished in endothelium vascular rings treated with ODQ (an inhibitor of soluble guanylyl cyclase). Therefore, it is highly unlikely that MAE activated soluble form of guanylyl cyclase in smooth muscle directly for vasorelaxation. The possibility that endothelium-derived vasorelaxant factors derived from cyclooxygenase involved in the vasorelaxant properties of MAE is unlikely since the endothelium-dependent relaxation caused by MAE in aortic rings was not affected by indomethacin.

However, the above-mentioned findings were not enough to rule out the possibility that MAE could also mediate vasorelaxation via the involvement of other receptors/mechanisms. Calcium (Ca^2+^) plays a significant role in the synthesis or release of NO from endothelial cells [28, 29]. It has been demonstrated that endothelium-mediated vasodilators cause an increase in inositol triphosphate (IP3) mediated Ca^2+^ release from intracellular stores which subsequently leads to the opening of Ca^2+^ dependent potassium (K^+^) channels in endothelial cells [30, 31].

Opening of the Ca^2+^ dependent K^+^ channels increases K^+^ efflux, hyperpolarizes vascular endothelial cells, and thus provides the driving force for Ca^2+^ influx into endothelial cells. In the present study, the endothelium-dependent relaxation to MAE was attenuated by TEA, a nonselective K^+^ channel blocker. These findings demonstrate that MAE might activate K^+^ channels in vascular endothelial cells, which results in an influx of Ca^2+^ and subsequent activation of the endothelial nitric oxide synthase (eNOS). Taken together, the present findings indicate that MAE causes potent endothelium-dependent relaxations involving, at least in part, NO/cGMP pathway and activation of K^+^ channel.

Endothelium-derived NO seems to play pivotal roles in vascular endothelium such as maintenance of vascular tone, modulation of the growth of VSMCs, and decrease in platelet adhesion/aggregation [32, 33]; therefore, impaired NO production is closely linked to the endothelial dysfunction or injury, which is considered to be a crucial factor in increasing risk of CVDs [29, 34]. Similarly, endothelium-dependent vasorelaxation is an important physiological determinant of normal endothelial cell function; however, an impaired endothelial function may be involved in the development of atherosclerosis. Therefore, the identification of plant extracts with the ability to increase the production of NO by vascular endothelium can be of great importance for the prevention and treatment of these CVDs.

Additionally, under different aspect we performed another experiment. VSMCs proliferation and migration play key role in provoking restenosis and atherosclerosis, so inhibition of VSMCs proliferation and migration is main target to control atheroma progression [35, 36]. For* in vitro* experiment, PDGF-BB was used to induce proliferation and chemoattractant for migration of VSMCs as notable evidence supports the fact that PDGF-BB can remarkably induce VSMCs migration and proliferation [37, 38]. In our* in vitro *model, MAE significantly inhibited PDGF-BB induced VSMCs proliferation and migration in a dose dependent manner. Our current study has provided further mechanistic insight to the vasoprotective effect of MAE and might help explain its beneficial effect on the cardiovascular system. Future cardiovascular research involving* Morus alba* root bark extract should include the detailed mechanisms of action, specificity, structure and function relationship, and therapeutic studies in both animal models and human trials.

Recent studies have indicated that most blood vessels are embedded in, or surrounded by, perivascular adipose tissue (vessel supporting connective tissue) that play important role of vascular homeostasis including maintenance of vascular function and progression of atherosclerosis [39, 40]. The outer adventitial layer is a collagen-rich connective tissue containing adipocytes as well as perivascular nerves [41]; therefore, during cleaning of connective tissues for vascular reactivity study, the aortic wall may also be devoid of some of its components which play a crucial role in vascular physiology [40–42]. Authors in this study acknowledge the limitation of this research of not using vessels with complete vascular wall as control. Taken together, further studies concerning the effect of MAE on the perivascular fat and adventitia will provide the more detail evidences of the protective effect of MAE in vasculature.

## 5. Conclusion

Our research revealed that* Morus alba* root extract is a potent endothelium-dependent vasodilator in part through endothelial-dependent NO-cGMP pathway including the activation of TEA sensitive K^+^ channels. In addition, MAE attenuated PDGF-BB induced VSMCs proliferation and migration. This study provides a novel insight to the cardioprotective effect of MAE and reinforces use of this plant with medicinal value for improving cardiovascular health associated with endothelial dysfunction.

## Data Availability

The data used to support the findings of this study are available from the corresponding author upon request.

## Abbreviations

PDGF: Platelet derived growth factor
VSMCs: Vascular smooth muscle cells
MTS: 3-(4,5-Dimethylthiazol-2-yl)-5-(3-carboxymethoxyphenyl)-2-(4-sulfophenyl)-2H-tetrazolium
MAE: Root bark extract of* M. alba*
NO: Nitric oxide
cGMP: Cyclic-guanosine monophosphate
CVDs: Cardiovascular disease
TNF-*α*: Tumor necrosis factor alpha
K^+^: Potassium
L-NA: N*ω*-Nitro-L-arginine
ODQ: 1*H*-[1,2,4] oxadiazole [4,3-a] quinoxalin-1-one
TEA: Tetraethylammonium
INDO: Indomethacin
FBS: Fetal bovine serum
EDR: Endothelium-dependent relaxations
Ca^2+^: Calcium
eNOS: Endothelial nitric oxide synthase
IP_3_: Inositol triphosphate.
